# Comparative study of the efficacy and tolerability of dihydroartemisinin - piperaquine - trimethoprim *versus *artemether - lumefantrine in the treatment of uncomplicated *Plasmodium falciparum *malaria in Cameroon, Ivory Coast and Senegal

**DOI:** 10.1186/1475-2875-10-185

**Published:** 2011-07-08

**Authors:** Hervé Menan, Oumar Faye, Albert Same-Ekobo, Agbaya Serge S Oga, Babacar Faye, Christiane P Kiki Barro, Thomas Kuete, Jean-Louis N'diaye, Ama-Moor Vicky, Rogert Tine, William Yavo, Dieynaba Kane, Kondo F Kassi, Moussa Kone

**Affiliations:** 1 Department of Parasitology, Faculty of Pharmacy, University of Cocody, Abidjan, Côte d'Ivoire; 2 Department of Public Health, Faculty of Pharmacy, University of Cocody, Abidjan, Côte d'Ivoire; 3 Department of Parasitology, Faculty of Medicine, University Cheikh Anta Diop, Dakar, Sénégal; 4 Hospital University Center of Yaoundé, Cameroun; 5 FMSP. University of Douala, Cameroun; 6 FMSB. University of Yaoundé 1, Cameroun

## Abstract

**Background:**

The ACT recommended by WHO is very effective and well-tolerated. However, these combinations need to be administered for three days, which may limit adherence to treatment.

The combination of dihydroartemisinin - piperaquine phosphate - trimethoprim (Artecom^®^, Odypharm Ltd), which involves treatment over two days, appears to be a good alternative, particularly in malaria-endemic areas. This study intends to compare the efficacy and tolerability of the combination dihydroartemisinin - piperaquine phosphate - trimethoprim (DPT) versus artemether - lumefantrine (AL) in the treatment of uncomplicated *Plasmodium falciparum *malaria in Cameroon, Ivory Coast and Senegal.

**Methods:**

This was a randomized, controlled, open-label clinical trial with a 28-day follow-up period comparing DPT to AL as the reference drug. The study involved patients of at least two years of age, suffering from acute, uncomplicated *Plasmodium falciparum *malaria with fever. The WHO 2003 protocol was used.

**Results:**

A total of 418 patients were included in the study and divided into two treatment groups: 212 in the DPT group and 206 in the AL group. The data analysis involved the 403 subjects who correctly followed the protocol (*per protocol *analysis), i.e. 206 (51.1%) in the DPT group and 197 (48.9%) in the AL group. The recovery rate at D14 was 100% in both treatment groups. The recovery rate at D28 was 99% in the DPT and AL groups before and after PCR results with one-sided 97.5% Confidence Interval of the rates difference > -1.90%. More than 96% of patients who received DPT were apyrexial 48 hours after treatment compared to 83.5% in the AL group (p < 0.001). More than 95% of the people in the DPT group had a parasite clearance time of 48 hours or less compared to approximately 90% in the AL group (p = 0.023). Both drugs were well tolerated. No serious adverse events were reported during the follow-up period. All of the adverse events observed were minor and did not result in the treatment being stopped in either treatment group. The main minor adverse events reported were vomiting, abdominal pain and pruritus.

**Conclusion:**

The overall efficacy and tolerability of DPT are similar to those of AL. The ease of taking DPT and its short treatment course (two days) may help to improve adherence to treatment. Taken together, these findings make this medicinal product a treatment of choice for the effective management of malaria in Africa.

## Background

The use of artemisinin-based combination therapy (ACT) is one of the strategies recommended by the WHO to effectively combat chemoresistance of strains of Plasmodium [1]. Artemisinins rapidly and effectively reduce the parasite biomass. In addition, very few documented cases of resistance to these compounds have been reported [2]. Because of their short half-life time, however, cases of parasite reinfection have occurred during their use in monotherapy to treat malaria. This also carries a risk of selecting chemoresistant strains of Plasmodium. Artemisinins need, therefore, to be associated with effective anti-malarials which have a relatively long half-life time. Not all combinations are good alternatives. This applies to the combination of artesunate/sulphadoxine-pyrimethamine (SP) because of the relatively limited effectiveness of SP in monotherapy [3,4] and this combination is not recommended in areas where the SP cure rate is under 80% [1]. In contrast, the combinations artesunate-mefloquine and artesunate-amodiaquine are highly effective, although they have disadvantages associated with their co-administration and tolerability.

The fixed-dose ACT artemether-lumefantrine (Coartem^®^) remains very effective and well tolerated. It is recommended, however, that it be administered twice daily for three days with a fat-rich meal, which may limit adherence to treatment. In this context, the combination of dihydroartemisinin - piperaquine phosphate - trimethoprim (Artecom^®^, Odypharm Ltd), which involves treatment over two days, appears to be a good alternative, particularly in malaria-endemic areas. This triple combination may also be a response to the reduced efficacy of dual ACT therapy that has already been seen in several Asian countries [5-9] and also a useful alternative in the face of increased selection of strains of Plasmodium resistant to the partner molecule of artemisinin in the combination [10]. No investigation had yet been conducted in Africa into the effectiveness and tolerability of Artecom^®^.

This study therefore intends to compare the efficacy and tolerability of the combination dihydroartemisinin - piperaquine phosphate - trimethoprim (DPT) versus artemether - lumefantrine (AL) in the treatment of uncomplicated *Plasmodium falciparum *malaria in Cameroon, Ivory Coast and Senegal.

## Methods

### Study sites

The study was conducted from September 2008 to February 2009 in three sub-Saharan African countries. Cameroon is located in central equatorial Africa and has a western sea front. Malaria is transmitted intensely in this country throughout the year, as it is in Ivory Coast, which occupies a central position in the Gulf of Guinea. In Senegal, located in the extreme west with its northern part on the edge of the Sahel and a western sea front, malarial transmission is seasonal with a peak between September and December.

### Study population

The study considered patients at least two years old suffering from acute, uncomplicated *Plasmodium falciparum *malaria with fever (axillary temperature ≥37.5°C) and a parasitaemia of between 1,000 and 100,000 trophozoites/μl in Senegal and 2,000 to 200,000 trophozoites/μl in Cameroon and Ivory Coast. Patients with signs of complicated malaria, severe malnutrition, repeated vomiting, intercurrent infectious disease, known allergy to the study drugs, past cardiac, hepatic or renal history or who were pregnant (positive test) or breast-feeding, were excluded.

Before inclusion, written informed consent was obtained from the patient or the patient's legal guardian. Approval was obtained from the national ethics committees in the three countries before the study was started. An insurance contract was also taken out in view of the possibility of serious adverse events.

### Study procedures

This was a randomized, controlled, open-label clinical trial with a 28-day follow-up period comparing DPT to AL as the reference drug. The WHO 2003 protocol was used [11]. Patients who were enrolled were weighed and randomized into each arm, either DPT or AL. In each study site computer generated randomization codes were prepared by an independent individual without the use of blocking. These codes were enclosed in sequentially numbered opaque sealed envelopes, each of which contained the treatment allocation. The envelopes were assigned in sequential order to participants after inclusion. The number of patients to be enrolled was determined by Epi Info 2000 software. The estimated expected recovery rate with AL was 98%, with a maximum acceptable difference of 5% to conclude that DPT was non-inferior and a power of 85%. The minimum number of people to be included in each arm was calculated from these assumptions to be 180 patients. Assuming a 10% loss to follow up the overall final target sample size of 400 participants was estimated.

DPT is presented in the form of tablets containing 32 mg of dihydroartemisinin, 320 mg of piperaquine phosphate and 90 mg of trimethoprim. The dose, which is administered in two divided daily doses, varies depending on patient weight: half a tablet mornings and evenings for two days from 10 to 19 kg, one tablet mornings and evenings for two days from 20 to 29 kg, one and a half tablet mornings and evenings for two days from 30 to 39 kg, and two tablets mornings and evenings for two days over 40 kg.

AL is presented in the form of tablets containing 20 mg of artemether and 120 mg of lumefantrine. This drug is also administered depending on the patient's weight on T0, T8, T24, T36, T48 and T60 h.

All of the daily doses were taken in the health centre with the assistance of a co-investigator. If the patient vomited within thirty minutes after the drug was administered, the whole dose was re-administered. If the vomiting persisted, the patient was excluded from the study and referred to the health centre doctor for management according to the current national policy. The dose could not be administered again if vomiting occurred more than 60 minutes after administration. If the patient could not tolerate the study drug or if his/her medical condition deteriorated, he/she was required to stop the treatment. The same approach was used for treatment failures. In this situation, a standard replacement anti-malarial therapy (quinine salts) was to be administered and recorded in the case report form. The patient was to continue being followed up, in line with the schedule.

Patients were examined clinically on D1, D2, D3, D7, D14, D21 and D28 after inclusion and thick and thin blood films were performed at each visit. The density of *P. falciparum *in the peripheral blood was determined by counting the number of asexual parasites in 200 white blood cells (WBC). All of thick and thin blood films were reread to double check. Haematological (full blood count) and biochemical (creatinine, transaminases and bilirubin) investigations were performed on D1 and D7. Genetic profiles for markers of the MSP1 and MSP2 polymorphisms were studied if the parasitaemia re-emerged as of D7 during the patient's follow-up, in order to distinguish reinfection from parasite recrudescence.

### End points

The primary end point was the recovery rate, defined as the percentage of patients who had an adequate clinical and parasitological response (ACPR) after follow-up for 28 days. Efficacy was evaluated using an intention to treat analysis which included all the 418 randomized patients then using a per protocol analysis which included the 403 patients who completed 28 days follow-up. The secondary end points were the incidence of early clinical failure (ECF), late parasitological failure (LPF), late clinical failure (LCF), change in gametocyte carrier status, abolition of fever and parasites and adverse clinical and laboratory events.

### Data analysis

Data were registered in Epi data version 3.1 software. The statistical analysis was carried out on Spss 12.0 software for windows. Demographic, clinical and laboratory parameters of the two groups at inclusion were compared using the t test for independent samples or Mann-Whitney test. A pure intention to treat analysis was performed using Kaplan-Meier survival analysis with log rank testing the treatment failures distribution function. Then, per protocol recovery rates were estimated and comparison of treatment efficacy was made using rates difference with one-sided 97.5% confidence interval. Fisher's exact test was used where appropriate. The distributions of fever and parasite clearance were compared using Pearson's Chi-2 test. Differences of haematological and biochemical parameters values within individuals between D1 and D7 were computed. Changes in haemoglobin concentrations and in other laboratory tolerability parameters were compared using the paired t test. The level of significance for statistical tests was set at 0.05.

## Results

### Global distribution of patients in the study

**Figure 1:**
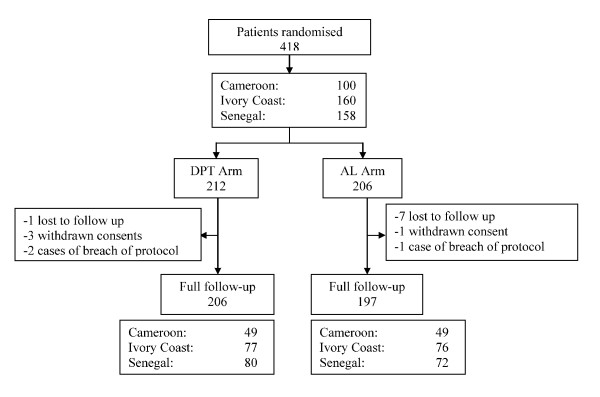
**Study profile showing the number of patients recruited into each arm**.

A total of 418 patients were included in the study and divided into two treatment groups: 212 in the DPT group and 206 in the AL group. The protocol was discontinued in 15 cases during the follow-up period, six in the DPT group and nine in the AL group (1 patient lost to follow-up, 3 withdrawals of consent and 2 refusals of blood draws in the DPT group and 7 patients lost to follow-up, 1 withdrawal of consent and 1 refusal of blood draws in the AL group). The study profile and distribution of patients in each treatment group and number of patients who underwent full follow-up are summarized in Figure 1. The data analysis involved the 403 subjects who correctly followed the protocol (*per protocol *analysis), i.e. 206 (51.1%) in the DPT group and 197 (48.9%) in the AL group.

### Demographic, fever, laboratory and clinical features of the patients in each arm

The demographic, fever, laboratory and clinical features of the patients are summarized in Tables 1 and 2 by treatment group. There were no statistically significant differences in distribution by sex or age band. Average temperatures in the two treatment groups were not statistically different. There were also no statistically significant differences between any of the laboratory parameters. Both groups were therefore statistically equivalent at inclusion.

**Table 1:** Comparison of the two treatment groups at inclusion

	DPT	AL	p*
	
Numbers	206	197	
Sex M, n *(%)*	97 *(47.1)*	93 *(47.2)*	*0.921*
F, n *(%)*	109 (*52.9)*	104 *(52.8)*	
**Mean age (SD) years**	15.51 (*12.64*)	15.58 (*11.91*)	*0.958*
**Min - Max**	2 - 60	2 - 62	
**[2 - 5], n *(%)***	36 *(17.5)*	30 *(15.2)*	
**[5 - 15], n *(%)***	87 *(42.2)*	85 *(43.1)*	
**[15-62], n *(%)***	83 *(40.3)*	82 *(41.6)*	

**Mean temperature (SD) °C**	38.73 (0.96)	38.58 (1.03)	*0.134*
**Min - Max**	35.5 - 41.0	35.2 - 41.4	
**< 37.5, n *(%)***	11 *(5.3)*	15 *(7.6)*	
**[37.5 - 38.5], n *(%)***	63 *(30.6)*	77 *(39.1)*	
**[38.5 - 41.4], n *(%)***	132 *(64.1)*	105 *(53.3)*	

**Median parasitaemia tpz/μl**	13225	13400	0.934**
**Min - Max**	1000 - 255000	1040 - 187500	

**Gametocyte carrier rate, n *(%)***	3 *(1.5)*	0 *(0)*	*0.249§*

**Mean ASAT (SD) IU/l**	30.59 (21.71)	31.36 (18.99)	*0.705*
**Min - Max**	5-165	7-134	

**Mean ALAT (SD) IU/l**	25.9 (20.61)	27.98 (16.20)	*0.262*
**Min - Max**	4-235	4-121	

**Mean creatinine (SD) mg/l**	8.28 (2.39)	8.26 (2.43)	*0.924*
**Min - Max**	3-20	4-20	

**Mean bilirubin (SD) mg/l**	9.57 (7.49)	10.88 (11.00)	*0.162*
**Min - Max**	2-59	2-85	

**Mean haemoglobin (SD) g/dl**	10.60 (1.81)	10.80 (1.85)	*0.289*
**Min - Max**	5.8-15.1	5.1-16.8	

* t test for independent samples **Mann-Whitney test § Fisher's exact test.

**Table 2:** Proportions of clinical signs at inclusion

Clinical signs	DPT N = 206, n *(%)*	AL N = 197, n *(%)*
**Fever**	195 (94.7)	182 (92.4)
**Headache**	164 (79.6)	153 (77.7)
**Asthenia**	105 (51.0)	103 (52.3)
**Anorexia**	89 (43.4)	76 (38.6)
**Rigors**	56 (27.2)	44 (22.4)
**Arthralgia**	72 (35.0)	69 (35.0)
**Abdominal pain**	40 (19.4)	56 (28.4)
**Pallor**	39 (18.9)	39 (19.8)
**Splenomegaly**	24 (11.7)	12 (6.1)
**Vomiting**	12 (5.8)	17 (8.6)
**Diffuse pain**	12 (5.8)	13 (6.6)
**Dizziness**	6 (2.9)	8 (4.1)
**Hepatomegaly**	6 (2.9)	3 (1.5)
**Nausea**	4 (1.94)	2 (1.0)
**Jaundice**	3 (1.5)	5 (2.5)
**Cough**	2 (1)	1 (0.5)
**Epigastric pain**	2 (1)	1 (0.5)
**Pruritus**	1 (0.5)	0 (0)

The median daily doses of the molecules were 2.5 mg/kg [1.4-4.7] for dihydroartemisinin, 7.1 mg/kg [3.8-13.3] for trimetoprime and 25.1 mg/kg [13.5-47.4] for piperaquine. They were 3.5 mg/kg [1.2-5.3] for artemether and 20.1 mg/kg [7.0-32.0] for lumefantrine.

### Efficacy of treatment

Kaplan-Meier estimates of recovery rates unadjusted by genotyping were 96.2% in the DPT group and 95.6% in the AL group. There was no statistical difference between the two groups (p = 0.755). The per protocol recovery rate at D14 was 100% in both treatment groups. The recovery rate [95% CI] at D28 was 99.0 [96.2 - 99.8]% in the DPT group and 99.0 [96.0 - 99.8]% in the AL group. These results are shown in Table 3. The rates difference was 0.04%, 97.5% CI > -1.90%.

**Table 3:** Distribution by response to treatment in each arm

	DPT n/N (%)	AL n/N (%)
**ACPR **(Adequate Clinical and Parasitological Response)	204/206 (99)	195/197 (99)
**ETF **(Early Treatment Failure)	0/206 (0)	1/197 (0.5)
**LCF **(Late Clinical Failure)	0/206 (0)	1/197 (0.5)
**LPF **(Late Parasitological Failure)	2/206 (1)	0/197 (0)
**Total Failure**	2/206 (1)	2/197 (1)

*p = 1 Fisher's exact test*.

Two cases of late parasitological failure (LPF) were seen in two patients in the DPT group at Day 28. Two cases of failure including one early treatment failure (ETF) at Day 4 and one late clinical failure (LCF) at Day 28 were seen in the AL group. PCR results confirmed the LPF and LCF. These were cases of parasitological recrudescence and not reinfection. The difference in response to treatment between the two groups was not statistically significant.

### Parasite clearance and abolition of fever

Fever was abolished within 24 hours in the majority of patients in both treatment groups. More than 96% (188/195) of patients who received DPT had no fever 48 hours after treatment compared to 83.5% (152/182) in the AL group. The difference between the distribution of times to abolish fever between the two groups was statistically significant (p < 0.001 Pearson's Chi-2 test).

**Figure 2:**
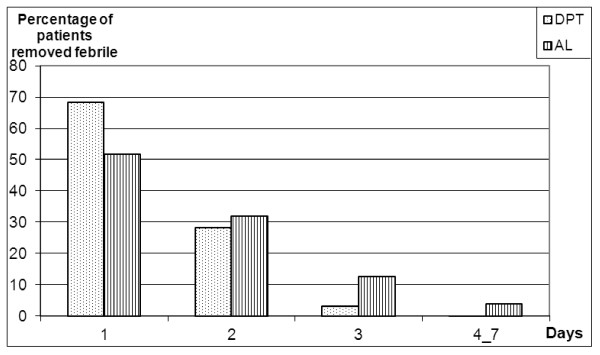
**Fever clearance**.

**Figure 3:**
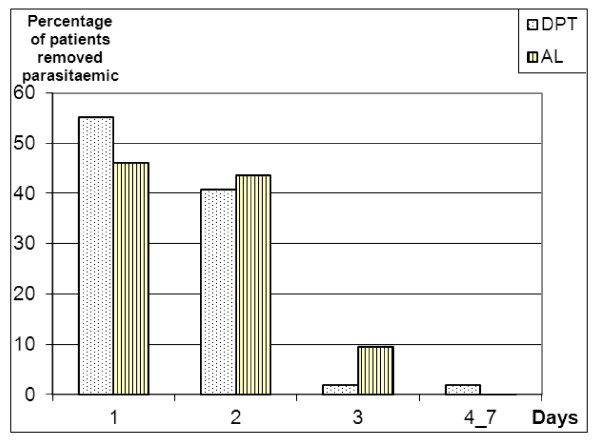
**Parasite clearance**.

More than 96% (198/206) of the people in the DPT group had a parasite clearance time of 48 hours or less compared to approximately 90% (177/197) in the AL group. The difference in distributions of times to achieve parasite clearance between the two groups was statistically significant (p = 0.023, *Pearson's Chi-2 test*). These features are summarized in Figures 2 and 3.

### Change in gametocyte carrier status

At inclusion, there were only three gametocyte carriers in the DPT group and none in the AL group. The number of gametocyte carriers fell to two on D2 and one on D3 in the DPT group. From D7 there were no more gametocyte carriers in this group. There were no patients who developed gametocytaemia after inclusion.

### Change in haemoglobin from D1 to D7

Examination of haemoglobin concentrations revealed a fall of individuals' values within the seven days after starting the study treatment. This fall in each of the two treatment groups was statistically significant (p < 0.001), as presented in Table 4. The fall of individuals' haemoglobin values summarized as mean of differences was greater in the AL group (1.0 g/dl) than in the DPT group (0.7 g/dl), p = 0.024. There were no statistical difference between study sites.

**Table 4:** Change in individuals' haemoglobin concentration by treatment group

	Mean haemoglobin concentration in g/dl (standard deviation)			p*
	
	D1	D7	D1-D7	
DPT	10.6 (1.82)	9.9 (1.65)	0.7 (1.38)	<0.001

AL	10.8 (1.9)	9.8 (1.8)	1.0 (1.28)	< 0.001

P**	0.289	0.570	0.024	

*paired t test. ** t test for independent samples

### Assessment of tolerability of DPT *versus *AL

Both drugs were therefore well tolerated and no serious adverse events were reported during the follow-up period. All of the adverse events observed were minor and did not result in the treatment being stopped in either treatment group. Ten cases of vomiting within 30 minutes were observed after administration of DPT and 13 cases after administration of AL also within 30 minutes. The whole dose was re-administered in each case.

The number of patients who experienced at least one adverse event in the DPT group, 47 (22.8%), was higher than those, 29 (14.7%), in the AL group, p = 0.038. 50 cases of adverse events were reported in the DPT group and 31 in the AL group (Table 5). The main adverse events reported were vomiting, abdominal pain and pruritus. It looks that vomiting was significantly more frequent in DPT group (10%) than AL group (3%), p = 0.004. The most of the adverse events after DPT were gastrointestinal. These side effects occurred more often in children than in adults.

**Table 5:** Incidence of adverse events reported

	DPT (N = 206) n *(%)*	AL (N = 197) n *(%)*
Vomiting*	21 *(10.2)*	6*(3)*
**Abdominal pain**	5 *(2.4)*	3 *(1.5)*
**Pruritus**	6 *(3)*	2 *(1)*
**Asthenia**	2 *(1)*	5 *(2.5)*
**Nausea**	4 *(2)*	3 *(1.5)*
**Dizziness**	1 *(0.5)*	4 *(2)*
**Epigastric pain**	3 *(1.5)*	0 *(0)*
**Diarrhoea**	2 *(1)*	0 *(0)*
**Skin rash**	2*(1)*	0 *(0)*
**Hypoglycaemia**	0 *(0)*	2*(1)*
**Insomnia**	0 *(0)*	2 *(1)*
**Facial oedema**	0 *(0)*	2 *(1)*
**Drowsiness**	2 *(1)*	0 *(0)*
**Aphthous ulcers**	0 *(0)*	1 *(0.5)*
**Skin fold lesions**	0 *(0)*	1 *(0.5)*
**Rash**	1 *(0.5)*	0 *(0)*
**Productive cough**	1 *(0.5)*	0 *(0)*
**Total**	50	31

* *p = 0.038, Pearson's Chi-2 test*

In terms of laboratory tolerability, individuals' values of transaminases fell in the DPT group whereas they rose in the AL group between the start of the study treatment and day seven. These differences, however, were not statistically significant. Similarly, serum creatinine fell between the start of treatment and day seven in the DPT group but rose in the AL group. The differences found in each group for this parameter, however, were not statistically significant. The fall in individuals' values of bilirubin between the start of the study treatment and day seven was statistically significant in both treatment groups but no statistical difference was found between treatment groups (Table 6).

**Table 6:** Changes in individuals' biochemical indices used to assess tolerability

Parameters	DPT				AL				P**
		
	D1	D7	D1-D7	p*	D1	D7	D1-D7	p*	
**ASAT** **(SD) IU/l**	30.53 (21.79)	28.83 (10.9)	1.7 (21.87)	0.271	31.25 (18.81)	32.69 (21.9)	-1.44 (28.52)	0.492	0.223

**ALAT** **(SD) IU/l**	25.75 (20.46)	25.74 (12.51)	0.01 (20.77)	0.997	28.32 (16.46)	28.63 (17.82)	-0.31 (23.94)	0.861	0.888

**Creatinine** **(SD) mg/l**	8.31 (2.39)	8.15 (2.46)	0.16 (2.59)	0.400	8.31 (2.46)	8.37 (2.47)	-0.06 (2.88)	0.799	0.430

**Bilirubin** **(SD) mg/l**	9.52 (7.56)	7.68 (4.11)	1.84 (8.74)	0.003	10.75 (11.03)	7.71 (3.69)	3.04 (11.68)	0.000	0.245

*paired t test.

** t test for independent samples comparing drop within individuals'values in DPT and AL groups.

## Discussion

In this study conducted in Cameroon, Ivory Coast and Senegal the two medicinal products, DPT and AL, were both effective in treating uncomplicated *P. falciparum *malaria. The recovery rates after follow up for 28 days were 99% for both medicinal products and the lower limit of the one-sided 97.5% confidence interval of the rates difference of -1.90%, up to the pre-established non inferiority margin of -5%, which allows us to confirm that DPT is not inferior to AL. In addition, abolition of fever and parasite clearance occurred faster in the DPT group than in the reference AL group.

However, these high recovery rates could have been overestimated due to the proportion of subjects over the age of 15, estimated at approximately 40% of the overall population of the study. This high proportion of subjects over the age of 15 in the study population is accounted for by that fact that many 2-5 year-old children were not included because one or more signs of severity of their malarial episode were discovered, whilst very few adults presented with severe signs. Furthermore, the 28-day protocol used in this study has limits in identifying cases of ETT [14].

This result was entirely expected, as the combination DP (dihydroartemisinin - piperaquine at doses of 20 mg and 160 mg in children and 40 mg and 320 mg in adults) had already been shown to have as effective anti-malarial activity as AL in several African studies [12-16]. Abolition of fever and parasite clearance were similar for DP and AL [17].

WHO currently recommends treatment with ACT for at least three days, and the recommended forms of ACT are only doublet combination therapies. However, the triple combination therapy suggested in the DPT combination appears to be an interesting alternative for reducing treatment time to two days, with excellent therapeutic efficacy and better parasite and thermal clearance times than those of AL.

Quadruple therapy, CV8, with a combination of primaquine and the three compounds in the studied combination, DPT, has been shown to offer excellent efficacy and good tolerability despite the number of associated anti-malarials [18]. The three compounds in DPT have completely different mechanisms of action and DPT was as well tolerated as AL. Whilst in terms of clinical tolerability, fewer adverse effects were seen in the AL group, it is important to note that the rise in transaminases was greater with this ACT. All adverse events were minor in both treatment groups and no treatment-related serious adverse events were seen.

One of the major benefits of the DPT combination is the reduction in treatment time, i.e. two days instead of three. However, the number of daily doses is two as against a single dose for some ACT such as DP. An improvement in the pharmaceutical formulation of the medicine with a view to reducing the number of daily dosings, particularly a single dosing, could be useful for improved observance of the anti-malarial treatment.

## Conclusion

The overall efficacy and tolerability of DPT are similar to those of AL with the advantage of a shorter treatment time of two days. The place of DPT in the armamentarium for the treatment of malaria must be further assessed to increase the efficacy of its use.

## List of abbreviations

DPT: combination Dihydroartemisinin - Piperaquine phosphate - Trimethoprim; AL: Artemether - Lumefantrine.

## Competing interests

The authors declare that they have no competing interests.

## Authors' contributions

OF, SE, KM supervised the clinical studies. HM, ASSOand YW analysed the data. All authors contributed to the drafting of the manuscript.
